# Do Improvements in Therapeutic Game-Based Skills Transfer to Real Life Improvements in Children's Emotion-Regulation Abilities and Mental Health? A Pilot Study That Offers Preliminary Validity of the *RET*hink In-game Performance Scoring

**DOI:** 10.3389/fpsyt.2022.828481

**Published:** 2022-03-21

**Authors:** Oana A. David, Silvia Magurean, Cristina Tomoiagă

**Affiliations:** ^1^Department of Clinical Psychology and Psychotherapy, Babeş-Bolyai University, Cluj-Napoca, Romania; ^2^The International Institute for the Advanced Studies of Psychotherapy and Applied Mental Health, Cluj-Napoca, Romania; ^3^Psychology Department, West University of Timişoara, Timişoara, Romania; ^4^Evidence-Based Psychological Assessment and Interventions Doctoral School, Babeş-Bolyai University of Cluj-Napoca, Cluj-Napoca, Romania

**Keywords:** serious games, children and adolescents, prevention, emotional disorders, digital health

## Abstract

Therapeutic or serious games are considered innovative ways of delivering psychological interventions especially suited for children and adolescents, which can have a positive impact on mental health, while also being fun and easily accessible online. While most serious games for children and adolescents address specific issues, such as anxiety or depression, preventive measures received less attention. *RET*hink is an online therapeutic game designed as a stand-alone prevention tool, aiming to increase resilience in healthy children and adolescents in a Rational Emotive Behavioral Therapy framework (David et al., 2019). The aim of this pilot study was to investigate the validity of in-game performance measurements or scores as indicators of the game effectiveness in building real life emotion-regulation abilities. We analyzed how scores of different game levels (addressing different skills) are associated with improvements in mental health and emotion regulation abilities. Our preliminary results suggest that in-game performance at some levels (scores) consistently reflect improvements in psychological functioning, while in-game performance at other levels are less associated with changes in real life self-reported psychological functioning. These results offer important information about which levels can be used as preliminary indicators of psychological improvements, and which levels need to be revised in terms of task or scoring. Overall, results of our study offer preliminary validation of *RET*hink's game scoring system, while also suggesting the elements to be refined.

## Introduction

In the past years, researchers‘ attempts to bring psychological interventions closer to individuals in need have led to the development of online therapeutic or serious games. Therapeutic games are designed to be attractive, fun, and motivating, while incorporating elements which trigger behavior and attitude change, pursue therapeutic goals, or train different skills (1, 2). A systematic review of the literature (3) regarding serious games as psychotherapeutic interventions observed positive effects on self-esteem, self-efficacy, knowledge, adherence to treatment, problem solving skills, as well as cognitive and behavioral aspects of aggression.

As Brezinka (4) noticed, serious games are innovative ways of delivering psychological interventions especially suited for children and adolescents. They are fascinated by technology and games, and serious games could provide an environment in which children would receive attractive homework assignments, would be able to rehearse skills or concepts acquired during therapy session, and all these could increase child compliance. Moreover, because they can be made available online, serious games can reach more children and adolescents who might otherwise not have access to psychological interventions.

Several therapeutic and serious games were developed as psychological tools for children and adolescents [e.g., *Treasure Hunt*, (4); *SPARX*, (5)], with promising effects on psychological symptoms [for a review see (2, 6)]. However, most of these games were developed to address specific issues, such as anxiety, depression, fear, or attention deficit. However, none of the previous efforts attempted using a therapeutic game as standalone prevention based on transdiagnostic model of emotional disorders. Thus, our approach was to investigate a therapeutic game aimed to improve emotional abilities of children and adolescents in order to prevent future problems of mental health.

In this context, *RET*hink game was designed to improve emotional skills in healthy children and adolescents, as a stand-alone preventive program for mental health (7–9). *RET*hink was developed in a Rational Emotive Behavioral Therapy framework [REBT; (10)] and its preventive program Rational Emotive Behavior Education [REBE; (11)], meant to teach children emotion awareness and cognitive change skills (i.e., identify their irrational beliefs, and replace them with their alternative rational beliefs). *RET*hink was investigated in a randomized clinical trial and found to have a preventive effect in healthy children by reducing emotional symptoms and depressive mood, while increasing their emotional regulation ability. It was also found that changes in youths' irrational beliefs worked as mechanisms for helping them improve depressed mood (8).

The present article is a secondary analysis of the main study regarding the effectiveness of the *RET*hink game (7). The aim of this study was to investigate the validity of in-game performance measurements or scores as indicators of the game effectiveness in building emotion-regulation abilities. Throughout the seven levels of *RET*hink, participants have various tasks for helping the main positive character (RETMAN) save the Earth from the negative character (*Irrationalizer*). In each level, players have a specific mission through which they can conquer the Earth territories which were previously occupied by *Irrationalizer*. At the end of each level, players have to win the key which will allow them to access the next territory (level). Players‘ performance is registered by both number of errors (vs. correct actions), and by a total score, which is computed by different algorithms depending on the task for each level (as shown in Table 1).

**Table 1:** Short description of the *RET*hink levels and scoring algorithm.

**Level and short description**	**Scoring algorithm**
Level 1 Task: Recognizing base emotions, functional vs. dysfunctional negative emotions (part of the emotional-regulation process)	Gain 40 points for each correct action (in distinguishing between emotions); lose 20 points for each wrong action (in distinguishing between emotions). Maximum score: 121 emotions*40 points = 4,840. ^*^Repeating level in case of 25% of consecutive wrong answers or 50% wrong answers.
Level 2 Task: Recognizing irrational and rational beliefs (ability used to identify personal beliefs and change the irrational ones in order to have better regulate mood)	Gain 15 points for each correct action (cleaning tree from irrational singing birds/leaving rational singing birds/caring for the tree); lose 15 points for each wrong action.
Level 3 Task: Cultivating functional emotions (part of the emotional-regulation process)	Gain 5 points for each correct action (cultivating good seeds, caring for the good seeds); lose 5 points for each wrong action (cultivating bad seeds); lose 1 point per each seed bought
Level 4 Task: Matching irrational beliefs with rational beliefs (ability used to change the irrational beliefs in rational ones)	Gain 40 points for each correct action (in neutralizing each type of irrational belief with its corresponding rational belief); lose 40 points for each wrong action (in neutralizing each type of irrational belief with its corresponding rational belief). Each neutralizing potion bought costs 20 points.
Level 5 Task: Problem solving (trained ability to use in real-life situation)	Gain 50 points for each minute bought Lose 40 points for each minute bought and obstacle eliminated
Level 6 Task: Relaxation (important for improving mental health and part of the emotion-regulation abilities)	Lose 100 points for being detected by the Irrationalizer's team Successfully terminate level in case of 10 measurements under baseline without being detected
Level 7 Task: Matching rational beliefs and happiness with needs (important for improving mental health) *Due to a technical issue, the scores for level 7 were erroneously registered by the software*	Sublevel 7.1: Build positive attention bias: Earn 50 points for each correct action (identify the happy face); lose 50 points for each wrong action (identify the happy face); lose 40 points if time is up. Sublevels 7.2 and 7.3: Earn 40 points for each correct action (matched rational belief/happiness need); lose 40 points for each wrong action (unmatched rational belief/happiness need); lose 40 points for failing to embrace happiness. Sublevel 7.2: Maximum score: 17 characters*40 points=680 points Sublevel 7.3: Maximum score: 21 characters*40 points = 840 points

* *is a specification added to the scoring of the level*.

We conducted a pilot study with a small sample size and for the purpose of this study, we used the total score for each level as an indicator for game-based skills. We also used delta changes between two consecutive play sessions as indicator of game-based gains in emotion-regulation abilities.

Demonstrating the validity of in-game indicators as markers of game effectiveness in building real life self-reported emotion-regulation skills could prove advantageous for several reasons. First, analyzing how each game level, based on in-game tasks and exercises bring specific real-life psychological benefits is informative for the game efficacy and could help improve the game scoring, and the specific tasks within the seven levels of the game. Second, the in-game indicators could provide insights into how much a child benefits from the game, without having formal and complex psychological assessments. This would support the use of the *RET*hink therapeutic game as stand-alone application and making sure that its scoring matches ability gains for motivational purposes.

## Objectives

For the purpose of this pilot study we investigated how the *RET*hink game-based skills are translate in real life improvements, consisting of reported mental health and emotion-regulation skills. Our specific expectations/hypotheses for each game level performance/score are described below:

- Higher game-based emotion recognition skills (Higher scores at Level 1) will be correlated with higher gains in self-reported mood (measured by SDQ, EATQ, FD-CMS) and emotion-regulation abilities (measured by ERICA).
- Higher game-based skills in recognizing irrational processes (Higher scores at Level 2, 3) and changing them (Higher scores at Level 4) will be correlated with higher improvements (gains) in self-reported irrational/rational beliefs (measured by CASI).
- Higher game based problem-solving skills (Higher scores at Level 5) will be correlated with higher improvements in self-reported problem-solving (measured by VAS).
- Higher game based relaxation skills (Higher scores at Level 6) will be correlated with higher improvements in self-reported negative mood and stress (measured by FD-CMS).

Our secondary aim was to investigate if gains in performance from consecutively playing the *RET*hink game are associated with real life self-reported improvements in in mental health and emotion-regulation skills. Thus, we expected that improvements in skills at each level (higher scores) are related to self-reported real life improvements in mental health and emotion regulation skills.

## Methods

### Participants

Children and adolescents (*N* = 54) assigned to the *RET*hink condition in the clinical trial by David et al. (7) represent the sample used for the pilot study. The final sample consisted of 48 children and adolescents (six participants failed to complete the initial assessment and were treated as dropouts). Most participants were girls (*N* = 36), and their age ranged between 10 and 16 years, with a mean age of 13 years (*SD* = 2.05). Considering the small sample size our study is underpowered but being a pilot study can offer preliminary evidence for the effect of the game on self-reported improvements in real life. Written informed consent was obtained from the parents and the school management and the study was approved by the ethical committee of the institution.

### Procedure

#### *RET*hink Game

*RET*hink is a therapeutic game designed as an iOS application for building resilience in children and adolescents. The main goal of the game is to lead the positive character, RETMAN, and his rational friends in their quest of helping the people on Earth against the negative character, *Irrationalizer*, and his irrational servants. The five rational friends of RETMAN represent rational beliefs as follows: *Preferilizer* (representing preferences beliefs), *Ponderancer* (representing non-awfulizing beliefs), *Toleraser* (representing high frustration tolerance beliefs), *Acceptableizer* (representing unconditional acceptance beliefs) and *Optimizer* (representing happiness). The four irrational servants of Irrationalizer symbolize irrational beliefs: *Necessitizer* (representing demandingness beliefs), *Awfulizer* (representing awfulizing beliefs), *Frustralizer* (representing low frustration tolerance beliefs) and *Discourager* (representing global evaluation beliefs).

*RET*hink has seven levels which focus on objectives based on the REBT model: Level 1: identifying the emotional reactions, differentiating between basic emotions, complex emotions and functional and dysfunctional emotions, Level 2: identifying cognitive processes, Level 3: identifying the relation between cognitive processes, emotions and behavioral reactions, Level 4: changing irrational cognitions into rational cognitions, Level 5: building problem solving skills, Level 6: developing relaxation skills, and Level 7: consolidation of previous skills and building happiness skills (7).

For each level, RETMAN would make an introduction to explain the goal of the level and engage the player in a short trial (training) session before the actual level begun. For the actual game play, performance indicators were automatically registered during each level, and participants had to restart a level/sublevel if they did not finish 50% of the level, or had consecutive errors for 25% of the entire level/sublevel.

A short description of each level and scoring algorithm is presented in Table 1. For a detailed description of the game, see the studies regarding the development of *RET*hink and the effectiveness of the game (7, 8). When establishing the scoring algorithm we took under consideration relevant literature on the gaming and scoring topic [see (12, 13)].

During the clinical trial, participants in the *RET*hink group played each game twice, in order to consolidate skills developed throughout each level. The game sessions were organized in seven modules, each lasting ~50 min. The seven modules were delivered during 1 month, and participants played the game at school using Apple iPad Air 2 devices. As the first game session acts as a practice session, we only used for the analysis the game score for the second game session.

Because of a technical issue, the scores for the seventh level were erroneously registered by the software. Therefore, we excluded the seventh level scores from all the analyses.

Participants were subjected to three assessment sessions: a pre-intervention assessment, an intermediary assessment (after module 4), and a post-intervention assessment after the modules were completed. For the objective of the current study, we analyzed only the changes in psychological symptoms from the first to the last assessment.

### Measures

*Strengths and Difficulties Questionnaire—child version [SDQ;* *(**14**)]* is a 25-items self-report instrument measuring prosocial behavior as a psychological strength, and emotional symptoms, conduct problems, hyperactivity/inattention, and peer relationship problems as psychological difficulties. Higher scores for this instrument are representative for higher levels of psychological strength/difficulties. Internal consistencies found in the main study for SDQ are α = 0.75 for emotional symptoms subscale, α = 0.80 for the total level of psychological difficulties, α = 0.65 for conduct problems subscale, α = 0.65 for hyperactivity-attention subscale, α = 0.63 for peer problems subscale, and α = 0.67 for prosocial behavior subscale (7).

*The Early Adolescent Temperament Questionnaire—Revised [EATQ-R;* *(**15**)]* is a 65-items self-report questionnaire measuring temperamental effortful control, affiliativeness, surgency, and negative affectivity. For our study, we used only employed four dimensions of the instrument: depressive mood, attention, fear, and inhibitory control. Higher scores for each scale represent higher levels of the corresponding dimension. Reliability of the EATQ dimensions in the main study were α = 0.48 for attention subscale, α = 0.56 for fear subscale, α = 0.52 for inhibitory control subscale, and α = 0.64 for depressive mood subscale (7).

*The Emotion Regulation Index for Children and Adolescents [ERICA;* *(**16**)]* was designed as 17-item questionnaire addressing emotional-regulation in children and adolescents. ERICA measures three dimensions: emotional control, emotional self-awareness, and situational responsiveness. For each dimension, emotional regulation difficulties are represented by lower scores. In the main study, reliability for ERICA dimensions was α = 0.70 for emotional control subscale and α = 0.57 for emotional self-awareness subscale (7).

*The Child and Adolescent Scale of Irrationality [CASI;* *(**17**)]* is a 28-item scale designed to measure irrational cognitions in children and adolescents in several domains: demandingness for fairness (DEM-F), low frustration tolerance for work (LFTW), low frustration tolerance for rules (LFT-R), and the total irrationality score. Children and adolescents rated their agreement to the 28 sentences on a 5-point Likert scale, so that higher scores indicate high levels of each dimension. Internal consistency reported in the study regarding the mechanisms of change responsible for the effect of *RET*hink was α = 0.65 for low frustration tolerance for work, α = 0.80 for low frustration tolerance of rules, and α = 0.80 for CASI total score. The demandingness subscale showed lower reliability, α = 0.27 and was excluded from all analyses (8).

*Functional and Dysfunctional Child Mood Scales—girls and boys versions [FD-CMS;* *(**18**)]* contains 9 items on a 10-point Likert scale measuring intensity of emotions based on the binary model of distress (19). The instrument assesses the intensity of three types of emotions: functional negative emotions, dysfunctional negative emotions, and positive emotions. High scores for each scale indicates that the individual experienced the corresponding emotions at higher intensity. The measure registered adequate reliability in a preliminary study (18). Internal consistency obtained for FD-CMS in the entire sample included in the main study was α = 0.80 for functional negative emotions subscale, α = 0.65 for dysfunctional negative emotions subscale, and α = 0.66 for positive emotions subscale.

*Self-reported problem-solving* was assessed using a single item Visual Analog Scale with 10 levels.

## Results

### Data Analysis

Game performance is represented by the scores registered for each level during the second game session. Regarding changes in psychological symptoms, we computed delta change scores as the difference between the first and the final assessments. We performed normality tests and found normal distributions for our main variables. Considering our expectations regarding the association between game-based skills and improvements in real life self-reported functioning, the correlation analyses reported below are one-tailed. Because of technical issues, the score of the seventh game level was erroneously registered, therefore it was not included in the following analyses. Dropouts were removed from the analysis. Due to multiple testing and small sample size, we applied the Bonferroni correction in testing our four hypotheses, with a resulting *p* = 0.0125. For the predictive validity we performed regression analysis to assess if the scores at each level are predicting improvement in mental health. Means and standard deviations for the outcome variables are presented in Table 2.

**Table 2:** Means (M), standard deviations (SD), range (Min–Max) and sample size (*N*) for outcome variables.

	**Pre**				**Post**			
	**M**	**SD**	***n***	**Min-Max**	**M**	**SD**	***n***	**Min-Max**
CASI total	74.08	10.61	47	54.00; 101.00	65.64	17.52	48	32.00; 104.00
FDCMS total	6.62	10.83	48	0.00; 50.00	7.04	9.81	48	0.00; 41.00
SDQ total	20.38	5.02	47	12.00;31.00	18.70	5.62	48	11.00;30.00
ERICA control	26.78	5.46	47	13.00; 39.00	31.25	6.01	47	17.00; 40.00
ERICA awareness	19.02	3.08	47	13.00; 25.00	21.10	3.26	47	13.00; 25.00
EATQ attention	22.36	4.22	47	15.00; 34.00	25.37	4.56	48	17.00; 35.00
EATQ depression	15.06	4.67	47	7.00; 24.00	11.39	3.68	48	6.00; 21.00
EATQ inhibition	34.74	5.75	47	20.00; 45.00	37.79	6.83	48	23.00; 52.00
EATQ fear	16.04	4.80	47	7.00; 24.00	12.89	4.53	48	6.00; 22.00

*Higher game-based emotion recognition skills (Higher scores at Level 1) will be correlated with higher improvements in self-reported mood (SDQ, EATQ, FD-CMS) and emotion-regulation (ERICA)*.

**Figure 1:**
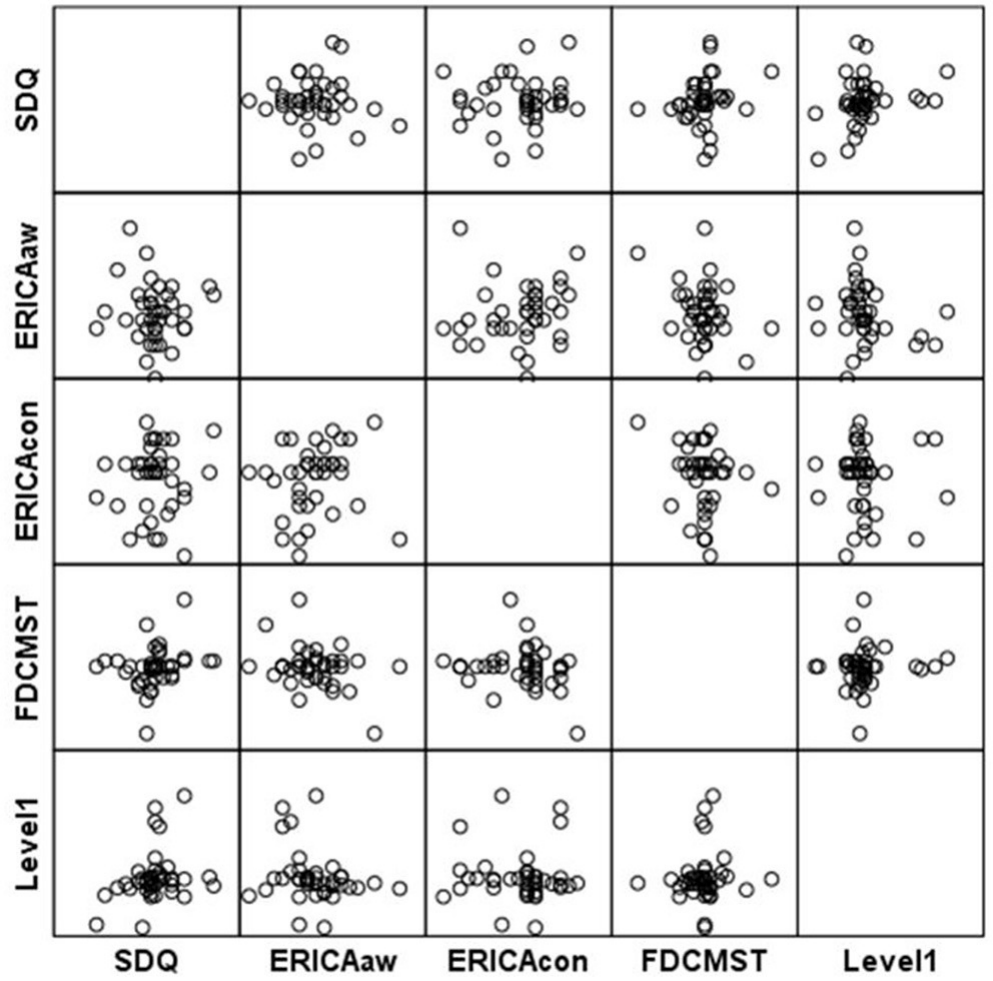
Scatterplot matrix for Level 1 scores.

**Figure 2:**
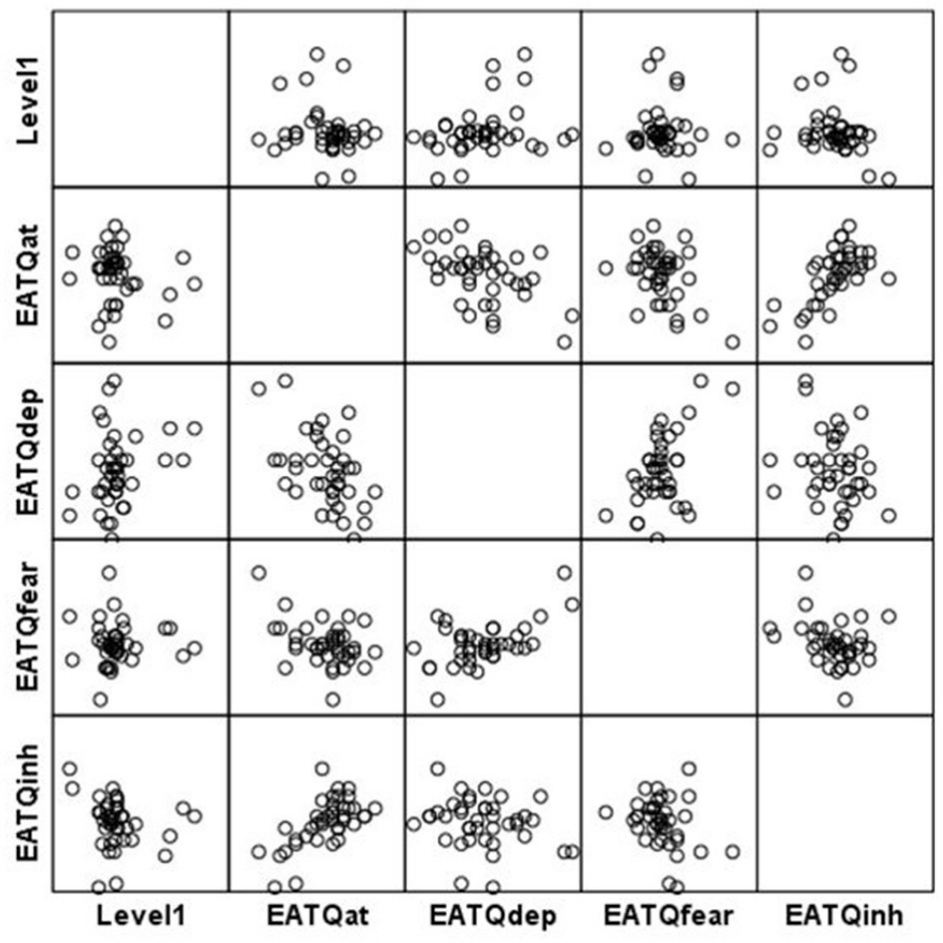
Scatterplot matrix for Level 1 scores.

Our results show that higher scores at Level 1 (game based emotion recognition skills) were correlated with improvements in mental health (SDQ) related conduct problems [*r*_(37)_ = 0.27, *p* = 0.04] and peer relations problems [*r*_(37)_ = 0.41, *p* = 0.004], as well as with improvements in the total score for strengths and difficulties experienced [*r*(_37)_ = 0.29, *p* = 0.03] (see Tables 3A,B and Figures 1, 2). Concerning improvements in psychological dimensions assessed by *Early Adolescent Temperament Questionnaire*, higher scores at Level 1 were correlated with improvements in the depressive mood [*r*_(38)_ = 0.29, *p* = 0.03]. Changes in attention, fear, and inhibitory control were not significantly associated with game-based emotion recognition skills (Level 1 performance). However, participants with higher emotion recognitions skills (Level 1 scores) reported experiencing significantly more positive emotions, as measured by FD-CMS [*r*_(38)_ = −0.38, *p* = 0.009]. Improvements in negative emotions (functional or dysfunctional), and emotion regulation were not significantly associated with game-based emotion recognition skills.

**Table 3A:** Correlation analysis for Level 1 scores.

		**Level 1**	**SDQ_**	**SDQ_**	**SDQ_**	**SDQ_**	**SDQ_**	**SDQ_**	**ERICA_**	**ERICA_**
		**scores**	**emot**	**behav**	**hiper_**	**prosocial**	**relation**	**total**	**awareness**	**control**
Level 1 scores	Pearson correlation	1	0.173	0.271^*^	0.135	−0.153	0.417^**^	0.296^*^	−0.172	−0.010
	Sig. (1-tailed)		0.146	0.047	0.207	0.176	0.004	0.034	0.144	0.475
	N	40	39	39	39	39	39	39	40	40
SDQ_emotion	Pearson correlation	0.173	1	0.485^**^	0.472^**^	−0.224	0.258^*^	0.787^**^	0.111	0.196
	Sig. (1-tailed)	0.146		0.000	0.000	0.065	0.040	0.000	0.231	0.096
	N	39	47	47	47	47	47	47	46	46
SDQ_behav	Pearson correlation	0.271^*^	0.485^**^	1	0.573^**^	0.004	0.403^**^	0.788^**^	0.047	−0.039
	Sig. (1-tailed)	0.047	0.000		0.000	0.489	0.002	0.000	0.378	0.398
	N	39	47	47	47	47	47	47	46	46
SDQ_hiper	Pearson correlation	0.135	0.472^**^	0.573^**^	1	−0.170	0.337^*^	0.809^**^	−0.157	−0.089
	Sig. (1-tailed)	0.207	0.000	0.000		0.126	0.010	0.000	0.149	0.278
	N	39	47	47	47	47	47	47	46	46
SDQ_prosocial	Pearson correlation	−0.153	−0.224	0.004	−0.170	1	−0.373^**^	−0.256^*^	0.285^*^	−0.140
	Sig. (1-tailed)	0.176	0.065	0.489	0.126		0.005	0.042	0.027	0.177
	N	39	47	47	47	47	47	47	46	46
SDQ_relation	Pearson correlation	0.417^**^	0.258^*^	0.403^**^	0.337^*^	−0.373^**^	1	0.609^**^	0.095	0.026
	Sig. (1-tailed)	0.004	0.040	0.002	0.010	0.005		0.000	0.264	0.432
	N	39	47	47	47	47	47	47	46	46
SDQ_total	Pearson correlation	0.296^*^	0.787^**^	0.788^**^	0.809^**^	−0.256^*^	0.609^**^	1	0.021	0.037
	Sig. (1-tailed)	0.034	0.000	0.000	0.000	0.042	0.000		0.444	0.404
	N	39	47	47	47	47	47	47	46	46
ERICA_awareness	Pearson correlation	−0.172	0.111	0.047	−0.157	0.285^*^	0.095	0.021	1	0.116
	Sig. (1-tailed)	0.144	0.231	0.378	0.149	0.027	0.264	0.444		0.220
	N	40	46	46	46	46	46	46	47	47
ERICA_control_	Pearson correlation	−0.010	0.196	−0.039	−0.089	−0.140	0.026	0.037	0.116	1
	Sig. (1-tailed)	0.475	0.096	0.398	0.278	0.177	0.432	0.404	0.220	
	N	40	46	46	46	46	46	46	47	47
FDCMST_disf	Pearson correlation	0.087	−0.045	0.045	0.188	0.335^*^	−0.142	0.025	−0.249^*^	−0.213
	Sig. (1-tailed)	0.296	0.382	0.382	0.103	0.011	0.171	0.435	0.046	0.075
	N	40	47	47	47	47	47	47	47	47
FDCMST_funct	Pearson correlation	0.083	0.199	0.139	0.435^**^	0.237	−0.022	0.275^*^	−0.132	−0.192
	Sig. (1-tailed)	0.306	0.089	0.176	0.001	0.055	0.441	0.031	0.188	0.098
	N	40	47	47	47	47	47	47	47	47
FDCMST_positive	Pearson correlation	−0.380^**^	−0.516^**^	−0.317^*^	−0.400^**^	0.282^*^	−0.338^*^	−0.537^**^	0.017	−0.020
	Sig. (1-tailed)	0.009	0.000	0.016	0.003	0.029	0.011	0.000	0.455	0.448
	N	39	46	46	46	46	46	46	46	46
FDCMST_total	Pearson correlation	0.095	0.106	0.111	0.369^**^	0.313^*^	−0.083	0.188	−0.206	−0.226
	Sig. (1-tailed)	0.279	0.240	0.230	0.005	0.016	0.290	0.103	0.083	0.063
	N	40	47	47	47	47	47	47	47	47
EATQ_atent	Pearson correlation	−0.132	−0.351^**^	−0.388^**^	−0.636^**^	0.033	−0.270^*^	−0.550^**^	0.065	0.133
	Sig. (1-tailed)	0.208	0.008	0.004	0.000	0.413	0.035	0.000	0.333	0.187
	N	40	46	46	46	46	46	46	47	47
EATQ_depres	Pearson correlation	0.294^*^	0.555^**^	0.287^*^	0.373^**^	−0.205	0.184	0.477^**^	−0.264^*^	−0.017
	Sig. (1-tailed)	0.033	0.000	0.027	0.005	0.086	0.110	0.000	0.036	0.455
	N	40	46	46	46	46	46	46	47	47
EATQ_fear	Pearson correlation	0.012	0.577^**^	0.330^*^	0.355^**^	−0.338^*^	0.267^*^	0.513^**^	−0.183	−0.114
	Sig. (1-tailed)	0.471	0.000	0.012	0.008	0.011	0.036	0.000	0.109	0.223
	N	40	46	46	46	46	46	46	47	47
EATQ_inhib	Pearson correlation	−0.210	−0.170	−0.260^*^	−0.184	0.063	−0.187	−0.254^*^	0.058	0.239
	Sig. (1-tailed)	0.097	0.129	0.041	0.110	0.338	0.107	0.044	0.349	0.053
	*N*	40	46	46	46	46	46	46	47	47

* *Correlation is significant at the 0.05 level (1-tailed)*.

** *Correlation is significant at the 0.01 level (1-tailed)*.

**Table 3B:** Correlation analysis for Level 1 scores.

		**EATQ_**	**EATQdepress**	**EATQ_**	**EATQinhib_**	**FDCMS**	**FDCMST_**	**FDCMSTpositive**	**FDCMST_**
		**atent**		**fear**		**disf**	**funct**		**total**
Level 1 scores	Pearson correlation	−0.132	0.294^*^	0.012	−0.210	0.087	0.083	−0.380^**^	0.095
	Sig. (1-tailed)	0.208	0.033	0.471	0.097	0.296	0.306	0.009	0.279
	N	40	40	40	40	40	40	39	40
SDQ_emotion	Pearson correlation	−0.351^**^	0.555^**^	0.577^**^	−0.170	−0.045	0.199	−0.516^**^	0.106
	Sig. (1-tailed)	0.008	0.000	0.000	0.129	0.382	0.089	0.000	0.240
	N	46	46	46	46	47	47	46	47
SDQ_behav	Pearson correlation	−0.388^**^	0.287^*^	0.330^*^	−0.260^*^	0.045	0.139	−0.317^*^	0.111
	Sig. (1-tailed)	0.004	0.027	0.012	0.041	0.382	0.176	0.016	0.230
	N	46	46	46	46	47	47	46	47
SDQ_hiper	Pearson correlation	−0.636^**^	0.373^**^	0.355^**^	−0.184	0.188	0.435^**^	−0.400^**^	0.369^**^
	Sig. (1-tailed)	0.000	0.005	0.008	0.110	0.103	0.001	0.003	0.005
	N	46	46	46	46	47	47	46	47
SDQ_prosocial	Pearson correlation	0.033	−0.205	−0.338^*^	0.063	0.335^*^	0.237	0.282^*^	0.313^*^
	Sig. (1-tailed)	0.413	0.086	0.011	0.338	0.011	0.055	0.029	0.016
	N	46	46	46	46	47	47	46	47
SDQ_relation	Pearson correlation	−0.270^*^	0.184	0.267^*^	−0.187	−0.142	−0.022	−0.338^*^	−0.083
	Sig. (1-tailed)	0.035	0.110	0.036	0.107	0.171	0.441	0.011	0.290
	N	46	46	46	46	47	47	46	47
SDQ_total	Pearson correlation	−0.550^**^	0.477^**^	0.513^**^	−0.254^*^	0.025	0.275^*^	−0.537^**^	0.188
	Sig. (1-tailed)	0.000	0.000	0.000	0.044	0.435	0.031	0.000	0.103
	N	46	46	46	46	47	47	46	47
ERICA_ awareness	Pearson correlation	0.065	−0.264^*^	−0.183	0.058	−0.249^*^	−0.132	0.017	−0.206
	Sig. (1-tailed)	0.333	0.036	0.109	0.349	0.046	0.188	0.455	0.083
	N	47	47	47	47	47	47	46	47
ERICA_ control	Pearson correlation	0.133	−0.017	−0.114	0.239	−0.213	−0.192	−0.020	−0.226
	Sig. (1-tailed)	0.187	0.455	0.223	0.053	0.075	0.098	0.448	0.063
	N	47	47	47	47	47	47	46	47
FDCMST_disf	Pearson correlation	−0.302^*^	0.211	0.004	−0.200	1	0.587^**^	−0.067	0.861^**^
	Sig. (1-tailed)	0.020	0.077	0.490	0.088		0.000	0.326	0.000
	N	47	47	47	47	48	48	47	48
FDCMST_func	Pearson correlation	−0.320^*^	0.320^*^	0.076	−0.188	0.587^**^	1	−0.315^*^	0.918^**^
	Sig. (1-tailed)	0.014	0.014	0.306	0.103	0.000		0.016	0.000
	N	47	47	47	47	48	48	47	48
FDCMST_postive	Pearson correlation	0.251^*^	−0.308^*^	−0.256^*^	0.269^*^	−0.067	−0.315^*^	1	−0.231
	Sig. (1-tailed)	0.046	0.019	0.043	0.035	0.326	0.016		0.059
	N	46	46	46	46	47	47	47	47
FDCMST_total	Pearson correlation	−0.350^**^	0.305^*^	0.050	−0.217	0.861^**^	0.918^**^	−0.231	1
	Sig. (1-tailed)	0.008	0.018	0.370	0.072	0.000	0.000	0.059	
	N	47	47	47	47	48	48	47	48
EATQ_atent	Pearson correlation	1	−0.326^*^	−0.348^**^	0.620^**^	−0.302^*^	−0.320^*^	0.251^*^	−0.350^**^
	Sig. (1-tailed)		0.013	0.008	0.000	0.020	0.014	0.046	0.008
	N	47	47	47	47	47	47	46	47
EATQ_depres	Pearson correlation	−0.326^*^	1	0.525^**^	−0.101	0.211	0.320^*^	−0.308^*^	0.305^*^
	Sig. (1-tailed)	0.013		0.000	0.250	0.077	0.014	0.019	0.018
	N	47	47	47	47	47	47	46	47
EATQ_fear	Pearson correlation	−0.348^**^	0.525^**^	1	−0.271^*^	0.004	0.076	−0.256^*^	0.050
	Sig. (1-tailed)	0.008	0.000		0.033	0.490	0.306	0.043	0.370
	N	47	47	47	47	47	47	46	47
EATQ_inhib	Pearson correlation	0.620^**^	−0.101	−0.271^*^	1	−0.200	−0.188	0.269^*^	−0.217
	Sig. (1-tailed)	0.000	0.250	0.033		0.088	0.103	0.035	0.072
	*N*	47	47	47	47	47	47	46	47

* *Correlation is significant at the 0.05 level (1-tailed)*.

** *Correlation is significant at the 0.01 level (1-tailed)*.

*Higher game-based skills in recognizing irrational processes (Higher scores at Level 2, 3) and changing them (Level 4) will be correlated with higher improvements in self-reported irrational/rational beliefs (CASI)*.

**Figure 3:**
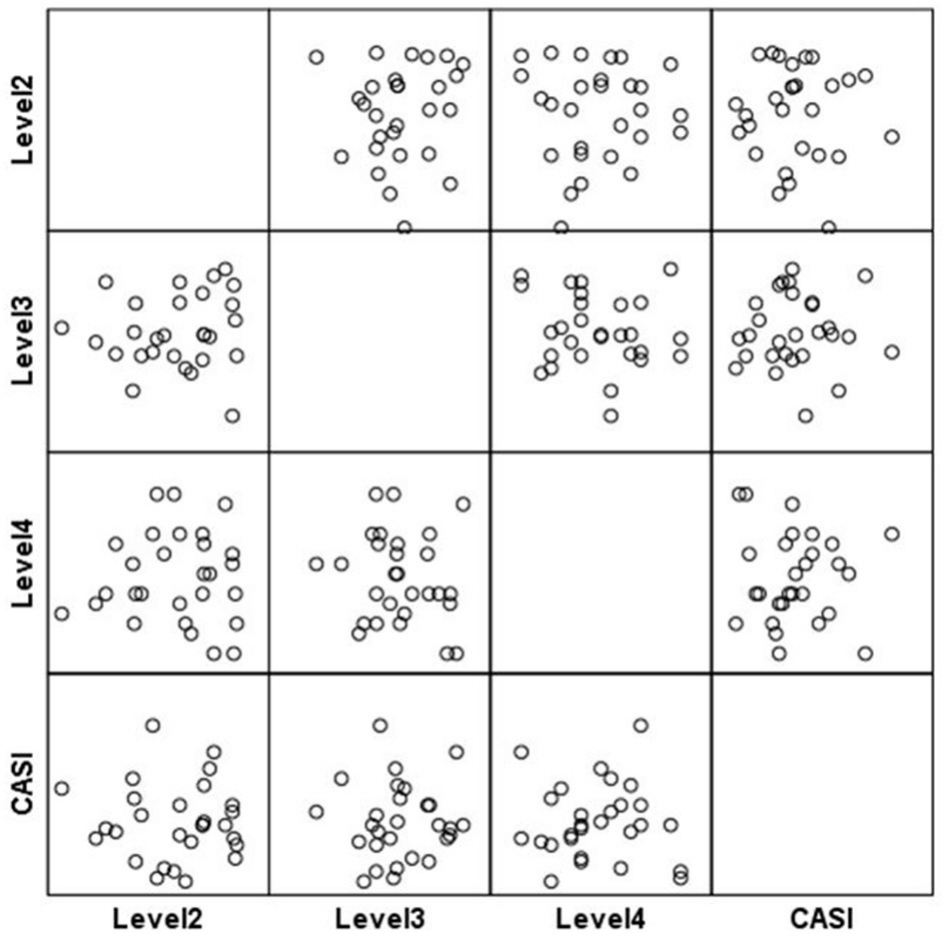
Scatterplot matrix for Level 2, 3 and 4 scores.

Higher game-based skills in recognizing and changing irrational processes were not reflected significantly in improvements in self-reported total irrational beliefs (see Table 4 and Figure 3). Participants with higher scores at Level 2 reported improvements in their frustration tolerance for rules [*r*_(34)_ = −0.29, *p* = 0.03], and increased their frustration tolerance for work [*r*_(34)_ = −0.24, *p* = 0.07], measured by CASI.

**Table 4:** Correlation analysis on Level 2, 3, and 4 scores.

		**Level 2 scores**	**Level 3 scores**	**Level 4 scores**	**CASI_ fair**	**CASI_ intoler**	**CASI_ work**	**CASI_ total**
Level 2 scores	Pearson correlation	1	0.172	−0.088	0.065	−0.298^*^	−0.242	−0.249
	Sig. (1-tailed)		0.169	0.322	0.354	0.039	0.077	0.071
	*N*	36	33	30	36	36	36	36
Level 3 scores	Pearson correlation	0.172	1	−0.174	−0.027	0.193	0.049	0.062
	Sig. (1-tailed)	0.169		0.159	0.432	0.111	0.379	0.347
	*N*	33	42	35	42	42	42	42
Level 4 scores	Pearson correlation	−0.088	−0.174	1	0.123	0.115	−0.069	0.149
	Sig. (1-tailed)	0.322	0.159		0.232	0.245	0.340	0.187
	*N*	30	35	38	38	38	38	38
CASI_fair	Pearson correlation	0.065	−0.027	0.123	1	0.083	0.218	0.262^*^
	Sig. (1-tailed)	0.354	0.432	0.232		0.289	0.071	0.037
	*N*	36	42	38	47	47	47	47
CASI_intoler	Pearson correlation	−0.298^*^	0.193	0.115	0.083	1	0.403^**^	0.766^**^
	Sig. (1-tailed)	0.039	0.111	0.245	0.289		0.002	0.000
	*N*	36	42	38	47	47	47	47
CASI_work	Pearson correlation	−0.242	0.049	−0.069	0.218	0.403^**^	1	0.750^**^
	Sig. (1-tailed)	0.077	0.379	0.340	0.071	0.002		0.000
	*N*	36	42	38	47	47	47	47
CASI_Total	Pearson correlation	−0.249	0.062	0.149	0.262^*^	0.766^**^	0.750^**^	1
	Sig. (1-tailed)	0.071	0.347	0.187	0.037	0.000	0.000	
	*N*	36	42	38	47	47	47	47

* *Correlation is significant at the 0.05 level (1-tailed)*.

** *Correlation is significant at the 0.01 level (1-tailed)*.

*Higher game based problem-solving skills (Higher scores at Level 5) will be correlated with higher improvements in self-reported problem-solving (VAS)*.

**Figure 4:**
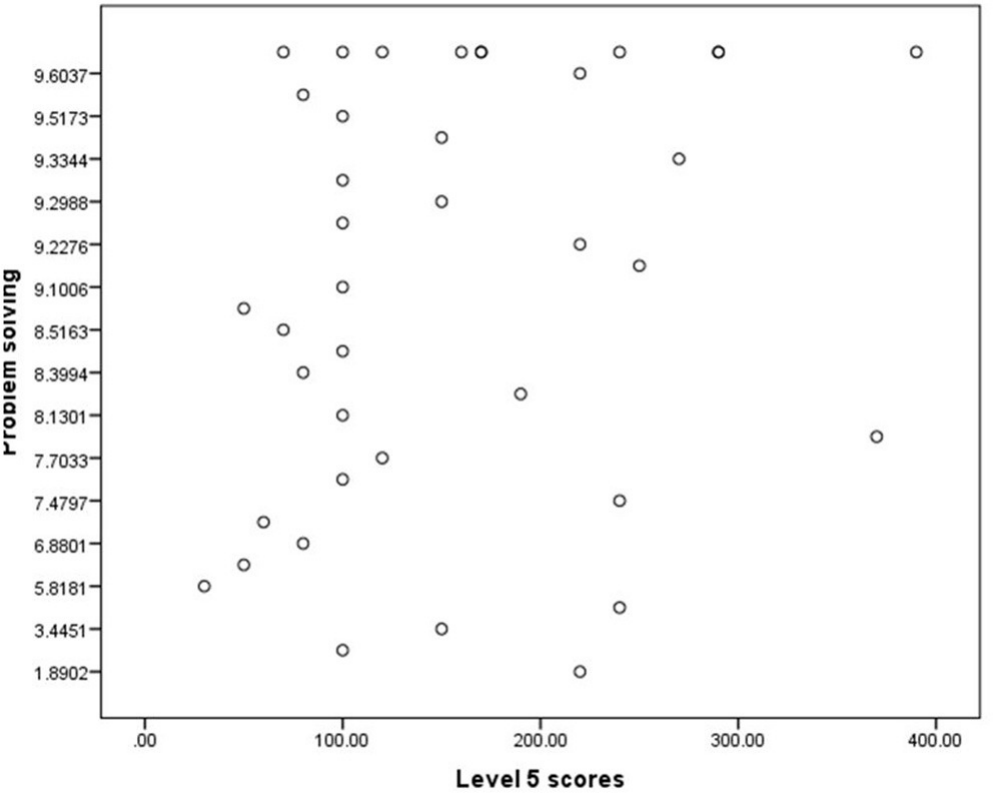
Scatterplot for Level 5 scores.

Our results suggest that self-reported problem solving was not significantly associated with the game based problem-solving skills [*r*_(37)_ = 0.14, *p* = 0.19] (see Table 5 and Figure 4).

**Table 5:** Correlation analysis for Level 5 scores.

		**Problem solving**	**Level 5 scores**
Problem solving	Pearson correlation	1	0.143
	Sig. (1-tailed)		0.193
	N	47	39
Level 5 scores	Pearson correlation	0.143	1
	Sig. (1-tailed)	0.193	
	N	39	39

*Higher game-based relaxation skills (Higher scores at Level 6) will be correlated with higher improvements in self-reported negative mood and stress*.

**Figure 5:**
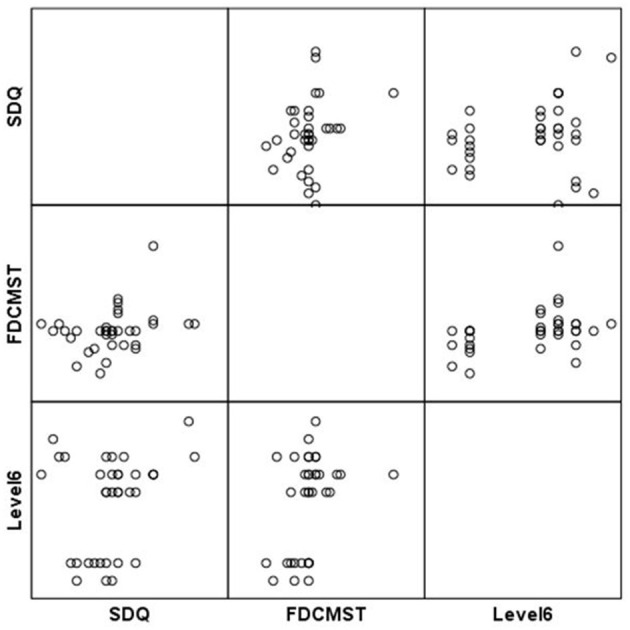
Scatterplot matrix for Level 6 scores.

**Figure 6:**
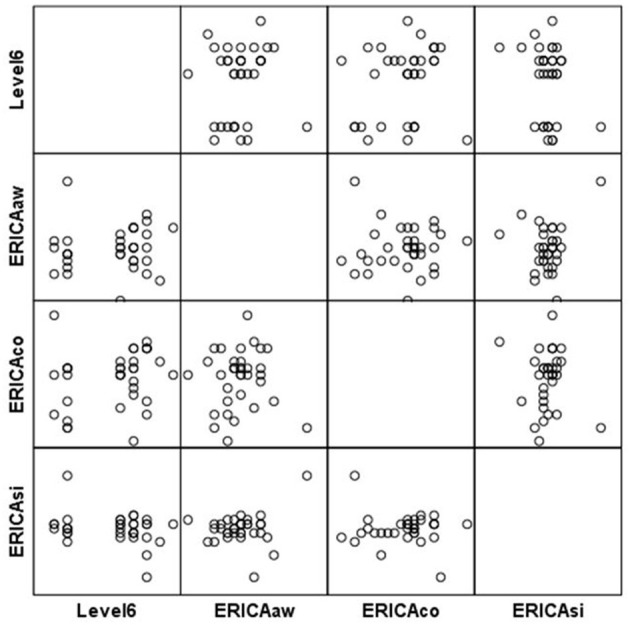
Scatterplot matrix for Level 6 scores.

Participants with higher scores at Level 6 reported significantly less functional negative emotions (FD-CMS) [*r*_(33)_ = 0.42, *p* = 0.006] and lower emotional difficulties [*r*_(33)_ = 0.37, *p* = 0.01], as measured by the SDQ (see Table 6 and Figures 5, 6). Moreover, higher game based relaxation skills were associated with a clear trend in improvements in emotional control [*r*_(33)_ = 0.22, *p* = 0.09] and situation responsiveness [*r*_(33)_ = −0.25, *p* = 0.06], measured by ERICA.

**Table 6:** Correlation analysis for Level 6 scores.

		**Level 6 scores**	**ERICA awarn**	**ERICA control**	**FDCMST disf**	**FDCMST funct**	**FDCMST positive**	**FDCMST total**	**SDQ emot**	**SDQ behav**	**SDQ hiper**	**SDQ prosocial**	**SDQ relation**	**SDQ total**	**ERICA situation**
Level 6 scores	Pearson correlation	1	0.082	0.223	0.203	0.424^**^	−0.117	0.397^**^	0.372^*^	0.076	0.184	0.237	0.105	0.256	−0.257
	Sig. (1-tailed)		0.320	0.099	0.121	0.006	0.255	0.009	0.014	0.332	0.145	0.085	0.275	0.069	0.068
	*N*	35	35	35	35	35	34	35	35	35	35	35	35	35	35
ERICA awareness	Pearson correlation	0.082	1	0.116	−0.249^*^	−0.132	0.017	−0.206	0.111	0.047	−0.157	0.285^*^	0.095	0.021	0.123
	Sig. (1-tailed)	0.320		0.220	0.046	0.188	0.455	0.083	0.231	0.378	0.149	0.027	0.264	0.444	0.205
	*N*	35	47	47	47	47	46	47	46	46	46	46	46	46	47
ERICA control	Pearson correlation	0.223	0.116	1	−0.213	−0.192	−0.020	−0.226	0.196	−0.039	−0.089	−0.140	0.026	0.037	−0.120
	Sig. (1-tailed)	0.099	0.220		0.075	0.098	0.448	0.063	0.096	0.398	0.278	0.177	0.432	0.404	0.211
	*N*	35	47	47	47	47	46	47	46	46	46	46	46	46	47
FDCMST disf	Pearson correlation	0.203	−0.249^*^	−0.213	1	0.587^**^	−0.067	0.861^**^	−0.045	0.045	0.188	0.335^*^	−0.142	0.025	0.320^*^
	Sig. (1-tailed)	0.121	0.046	0.075		0.000	0.326	0.000	0.382	0.382	0.103	0.011	0.171	0.435	0.014
	*N*	35	47	47	48	48	47	48	47	47	47	47	47	47	47
FDCMST funct	Pearson correlation	0.424^**^	−0.132	−0.192	0.587^**^	1	−0.315^*^	0.918^**^	0.199	0.139	0.435^**^	0.237	−0.022	0.275^*^	0.109
	Sig. (1-tailed)	0.006	0.188	0.098	0.000		0.016	0.000	0.089	0.176	0.001	0.055	0.441	0.031	0.234
	*N*	35	47	47	48	48	47	48	47	47	47	47	47	47	47
FDCMST positive	Pearson correlation	−0.117	0.017	−0.020	−0.067	−0.315^*^	1	−0.231	−0.516^**^	−0.317^*^	−0.400^**^	0.282^*^	−0.338^*^	−0.537^**^	0.066
	Sig. (1-tailed)	0.255	0.455	0.448	0.326	0.016		0.059	0.000	0.016	0.003	0.029	0.011	0.000	0.330
	*N*	34	46	46	47	47	47	47	46	46	46	46	46	46	46
FDCMST total	Pearson correlation	0.397^**^	−0.206	−0.226	0.861^**^	0.918^**^	−0.231	1	0.106	0.111	0.369^**^	0.313^*^	−0.083	0.188	0.226
	Sig. (1-tailed)	0.009	0.083	0.063	0.000	0.000	0.059		0.240	0.230	0.005	0.016	0.290	0.103	0.064
	*N*	35	47	47	48	48	47	48	47	47	47	47	47	47	47
SDQ emot	Pearson correlation	0.372^*^	0.111	0.196	−0.045	0.199	−0.516^**^	0.106	1	0.485^**^	0.472^**^	−0.224	0.258^*^	0.787^**^	−0.312^*^
	Sig. (1-tailed)	0.014	0.231	0.096	0.382	0.089	0.000	0.240		0.000	0.000	0.065	0.040	0.000	0.017
	*N*	35	46	46	47	47	46	47	47	47	47	47	47	47	46
SDQ behav	Pearson Correlation	0.076	0.047	−0.039	0.045	0.139	−0.317^*^	0.111	0.485^**^	1	0.573^**^	0.004	0.403^**^	0.788^**^	0.025
	Sig. (1-tailed)	0.332	0.378	0.398	0.382	0.176	0.016	0.230	0.000		0.000	0.489	0.002	0.000	0.435
	*N*	35	46	46	47	47	46	47	47	47	47	47	47	47	46
SDQ hiper	Pearson correlation	0.184	−0.157	−0.089	0.188	0.435^**^	−0.400^**^	0.369^**^	0.472^**^	0.573^**^	1	−0.170	0.337^*^	0.809^**^	−0.077
	Sig. (1-tailed)	0.145	0.149	0.278	0.103	0.001	0.003	0.005	0.000	0.000		0.126	0.010	0.000	0.305
	*N*	35	46	46	47	47	46	47	47	47	47	47	47	47	46
SDQ prosocial	Pearson correlation	0.237	0.285^*^	−0.140	0.335^*^	0.237	0.282^*^	0.313^*^	−0.224	0.004	−0.170	1	−0.373^**^	−0.256^*^	0.252^*^
	Sig. (1-tailed)	0.085	0.027	0.177	0.011	0.055	0.029	0.016	0.065	0.489	0.126		0.005	0.042	0.046
	*N*	35	46	46	47	47	46	47	47	47	47	47	47	47	46
SDQ relation	Pearson correlation	0.105	0.095	0.026	−0.142	−0.022	−0.338^*^	−0.083	0.258^*^	0.403^**^	0.337^*^	−0.373^**^	1	0.609^**^	−0.042
	Sig. (1-tailed)	0.275	0.264	0.432	0.171	0.441	0.011	0.290	0.040	0.002	0.010	0.005		0.000	0.390
	*N*	35	46	46	47	47	46	47	47	47	47	47	47	47	46
SDQ total	Pearson correlation	0.256	0.021	0.037	0.025	0.275^*^	−0.537^**^	0.188	0.787^**^	0.788^**^	0.809^**^	−0.256^*^	0.609^**^	1	−0.152
	Sig. (1-tailed)	0.069	0.444	0.404	0.435	0.031	0.000	0.103	0.000	0.000	0.000	0.042	0.000		0.157
	*N*	35	46	46	47	47	46	47	47	47	47	47	47	47	46
ERICA situation	Pearson correlation	−0.257	0.123	−0.120	0.320^*^	0.109	0.066	0.226	−0.312^*^	0.025	−0.077	0.252^*^	−0.042	−0.152	1
	Sig. (1-tailed)	0.068	0.205	0.211	0.014	0.234	0.330	0.064	0.017	0.435	0.305	0.046	0.390	0.157	
	*N*	35	47	47	47	47	46	47	46	46	46	46	46	46	47

** *Correlation is significant at the 0.01 level (1-tailed)*.

* *Correlation is significant at the 0.05 level (1-tailed)*.

For our second aim we perform regression analysis and results showed that only improvement at level 6 are predicting improvements in mental health (measured by Strengths and Difficulties Questionnaire) *p* = 0.036 (see Table 7).

**Table 7:** Regression analysis for predictive validity.

	**Unstandardized coefficients**		**Standardized coefficients**	***t***	**Sig**.
	**B**	**Std. error**	**Beta**		
(*Constant*)^a^	2.653	19.490		0.136	0.894
*Level 1 scores*	−5.011E-7	0.001	−0.000	−0.001	0.999
*Level 2 scores*	−0.001	0.009	−0.027	−0.115	0.911
*Level 3 scores*	−0.001	0.002	−0.075	−0.290	0.778
*Level 4 scores*	−0.020	0.023	−0.258	−0.890	0.395
*Level 5 scores*	0.012	0.022	−0.168	0.557	0.589
*Level 6 scores*	0.012	0.005	−0.579	2.417	0.036

a *Dependent Variable: Strengths and Difficulties Questionnaire (SDQ; 14)*.

## Discussion

The objective of the present pilot study was to analyze the in-game performance indicators of a preventive therapeutic game for children and adolescents (*RET*hink). Specifically, we aimed to investigate if these in-game performance indicators (scores for each level) are associated with improvements in real life self-reported mental health and emotion regulation skills. Demonstrating these associations could support the use of in-game scoring gains as marker of changes in psychological symptoms, thus allowing for a certain level of progress monitoring even outside the research lab, and without specialized psychological assessments.

Our preliminary analyses suggest that in-game performance at some levels (scores) consistently reflect improvements (statistically significant or plot trends) in psychological functioning, namely improvements—in total mental health, increased tolerance for rules, positive emotions, emotional control while in-game performance at other levels are less associated with changes in psychological functioning, namely reduced depressive mood, and lower emotional difficulties improvements.

Results obtained showed that improvements in-game scores at Level 1, which trains emotion recognition, are associated with self-reported improvements in youth's general mental health, as hypothesized. More specifically, higher level 1 game scores were associated with improvements in depressed mood, conduct problems and significantly with higher levels peer relationship problems and positive emotions. The association of emotion recognition in-game gains with real-life self-reported improvements in emotional abilities was in line with our expectation. We did not expect however specific significant improvements in conduct problems and peer relationships, which were surprising for us. It might be that by improving their emotional awareness and recognizing emotions in peers, children and adolescents were able to improve their aggressive behavior and their relationship problems. Future studies need to investigate if this is indeed the mechanism thought which emotional game abilities produced real life changes.

In terms of our second hypothesis regarding game scores at levels involving the recognition and change of irrational processes (Levels 2, 3, and 4), results obtained only partially confirmed our expectations. Children and adolescents who were better at identifying the connection between thinking and feeling based on their scores at Level 2, reported higher improvements in their irrational beliefs areas—more specifically in low frustration tolerance for work and rules. This is an important finding considering that the role of these specific irrational beliefs has been documented in relation to emotional difficulties and academic performance in children and adolescents (17). Results showed that youth with scores improvements in recognizing irrational processes and finding alternatives in game, were not necessarily the ones reporting improvements in their general irrational beliefs. This might be due to insufficient training of these specific skills during the game. However, since we have documented significant changes in irrational cognitions following the game (8) it might be that this signals rather the need to further calibrate the scoring for the Levels 3 and 4 of the game to reflect skills gain.

Our third hypothesis regarding improvements in scores obtained al Level 5 which trained problem solving skills being related to self-reported problem-solving abilities did not received support in our study. This might be due to the nature of the self-report measure which was a VAS type item. In future studies we need to employ more rigorous measures of problem solving abilities. Also, it might be that the tasks of the Level 5 need to be revised in order to better support problem-solving skills consolidation.

Results obtained provide support for our fourth hypothesis concerning positive associations between gains in game scores at Level 6, which trains relaxation abilities, and self-reported mood and stress. We found that children and adolescents that registered higher improvements in their in-game scores at this level, reported lower emotional difficulties, lower negative emotions and better emotion-regulation skills.

This study is not without limitations. First, due to technical difficulties we were not able to analyze if gains in the scores at Level 7 are related to real life improvements reported by children and adolescents. Future studies will need to investigate if scores obtained at this level are associated with mental health and positive emotionality. Second, our results need to be interpreted cautiously due to the small sample, lack of statistical power and gender disproportion which limited us in performing further analyses. Future studies need to include a larger sample in order to be able to delineate specific associations and draw clear conclusions regarding the predictive validity of the *RET*hink game scoring system. Third, future studies need to use more complex analyses in order to draw firm conclusions about the relationship between in-game scores and real-life improvements. Another limitation of the study are the measurements. We used self-report measures and some questionnaires, even though we found that they have good internal consistency on our sample, are not validated on the Romanian population. Future studies need to use other-report measures in addition to the self-report ones and use questionnaires that are validated for the population used.

In sum, our preliminary results showed that improvements in game scores are associated with improvements in self-reported mental health. More specifically, game scoring gains are associated with improved negative and positive emotions, conduct problems, peer relationship problems and emotion-regulation (especially at level 1, emotion recognition skills, and 6, relaxation skills). We have also found that better scores at Level 2 in recognizing the connection between thinking and feeling was associated with improvements in irrational thinking. These findings are in line with the findings from our clinical trial showing that the *RET*hink game is effective in promoting mental health by improving dysfunctional thinking mechanisms. Results of this study offer promising preliminary validation for the *RET*hink's game scoring and suggest that higher scores will reflect real-life changes in children and adolescents' mental health.

## Data Availability Statement

The raw data supporting the conclusions of this article will be made available by the authors, without undue reservation.

## Ethics Statement

The studies involving human participants were reviewed and approved by Ethical Committee of Babes-Bolyai University of Cluj-Napoca. Written informed consent to participate in this study was provided by the participants' legal guardian/next of kin.

## Author Contributions

OD contributed to the conception and design of the study. SM organized the database and performed the statistical analysis. OD, SM, and CT wrote the manuscript. All authors contributed to manuscript revision, read, and approved the submitted version.

## Funding

This work was supported by two grants awarded to OD from the Romanian National Authority for Scientific Research, CNCS—UEFISCDI (Grant Numbers PN-II-PT-PCCA-2013-4-1937 and PN-III-P4-ID-PCE-2020-2170). Trial Registration ClinicalTrials.gov, NCT03308981.

## Conflict of Interest

The authors declare that the research was conducted in the absence of any commercial or financial relationships that could be construed as a potential conflict of interest.

## Publisher's Note

All claims expressed in this article are solely those of the authors and do not necessarily represent those of their affiliated organizations, or those of the publisher, the editors and the reviewers. Any product that may be evaluated in this article, or claim that may be made by its manufacturer, is not guaranteed or endorsed by the publisher.
